# Adipose-Derived Mesenchymal Stem Cells Alleviate Hypertrophic Scar by Inhibiting Bioactivity and Inducing Apoptosis in Hypertrophic Scar Fibroblasts

**DOI:** 10.3390/cells11244024

**Published:** 2022-12-12

**Authors:** Shiyi Li, Jinxiu Yang, Jiachen Sun, Minliang Chen

**Affiliations:** Senior Department of Burns and Plastic Surgery, The Fourth Medical Center of Chinese PLA General Hospital, Beijing 100038, China

**Keywords:** adipose-derived mesenchymal stem cells, fibroblasts, oxidative stress, hypertrophic scar

## Abstract

**Background:** As a fibrotic disease with a high incidence, the pathogenesis of hypertrophic scarring is still not fully understood, and the treatment of this disease is also challenging. In recent years, human adipose-derived mesenchymal stem cells (AD-MSCs) have been considered an effective treatment for hypertrophic scars. This study mainly explored whether the therapeutic effect of AD-MSCs on hypertrophic scars is associated with oxidative-stress-related proteins. **Methods:** AD-MSCs were isolated from adipose tissues and characterized through flow cytometry and a differentiation test. Afterwards, coculture, cell proliferation, apoptosis, and migration were detected. Western blotting and a quantitative real-time polymerase chain reaction (qRT–PCR) were used to detect oxidative stress-related genes and protein expression in hypertrophic scar fibroblasts (HSFs). Flow cytometry was used to detect reactive oxygen species (ROS). A nude mouse animal model was established; the effect of AD-MSCs on hypertrophic scars was observed; and hematoxylin and eosin staining, Masson’s staining, and immunofluorescence staining were performed. Furthermore, the content of oxidative-stress-related proteins, including nuclear factor erythroid-2-related factor 2 (Nrf2), heme oxygenase 1 (HO-1), B-cell lymphoma 2(Bcl2), Bcl2-associated X(BAX) and caspase 3, was detected. **Results:** Our results showed that AD-MSCs inhibited HSFs’ proliferation and migration and promoted apoptosis. Moreover, after coculture, the expression of antioxidant enzymes, including HO-1, in HSFs decreased; the content of reactive oxygen species increased; and the expression of Nrf2 decreased significantly. In animal experiments, we found that, at 14 days after injection of AD-MSCs into human hypertrophic scar tissue blocks that were transplanted onto the dorsum of nude mice, the weight of the tissue blocks decreased significantly. Hematoxylin and eosin staining and Masson’s staining demonstrated a rearrangement of collagen fibers. We also found that Nrf2 and antioxidant enzymes decreased significantly, while apoptotic cells increased after AD-MSC treatment. **Conclusions:** Our results demonstrated that AD-MSCs efficiently cured hypertrophic scars by promoting the apoptosis of HSFs and by inhibiting their proliferation and migration, which may be related to the inhibition of Nrf2 expression in HSFs, suggesting that AD-MSCs may provide an alternative therapeutic approach for the treatment of hypertrophic scars.

## 1. Introduction

Hypertrophic scarring is a prevalent fibroproliferative disease in plastic surgery, and its incidence can be as high as 70% in burn patients [1]. However, the mechanism of hypertrophic scar formation is not yet precisely understood, and its prevention and treatment are relatively complicated. It is characterized by the excessive proliferation of dermal fibroblasts, resulting in the superabundant deposition of extracellular matrix (ECM) components such as collagen [2]. Current research shows that the pathogenesis of hypertrophic scarring is related to many signaling pathways or cytokines, such as the TGF-β/Smad pathway [3,4], the PI3K/AKT pathway [5], matrix metalloproteinases, the tissue inhibitors of metalloproteinases, and decorin [6].

Helmut [7] proposed oxidative stress in 1985. It refers to the pathological status in which the body’s excessive production of reactive oxygen species (ROS) or its reduced antioxidant capacity leads to a considerable accumulation of ROS and related products, bringing about many toxic effects on specific cells or molecules. Most of the intracellular ROS are derived from the oxidative respiratory chain, including the superoxide anion (O^2−^), hydrogen peroxide (H_2_O_2_), hydroxyl (OH^−^), etc., which have a particularly destructive effect on lipids, proteins, and nucleic acids [8,9]. Nuclear factor erythroid-2-related factor 2 (Nrf2) is the core antioxidant signal protein that can regulate the expression of a variety of antioxidant enzymes and apoptosis-related genes, such as superoxide dismutase (SOD), NAD(P)H: quinone oxidoreductase 1 (NQO1), heme oxygenase 1 (HO-1), and the B-cell lymphoma 2 protein (Bcl2) [10,11,12,13]. Under normal circumstances, Nrf2 binds to Kelch ECH associating protein 1 (KEAP1) and exists in the cytoplasm. When the ROS content in the cell increases, Nrf2 quickly dissociates from KEAP1, translocates into the nucleus, and regulates the expression of related factors [14]. Oxidative stress has been confirmed to be involved in the occurrence of many diseases, such as diabetes, coronary artery sclerosis, and hepatic fibrosis [10,15,16]. In 2009, De Felice et al. [17] found higher levels of ROS in hypertrophic scar fibroblasts (HSFs) and keloid fibroblasts (KFs) than in normal skin fibroblasts. Lee et al. [18] demonstrated the presence of higher protein carbonyl levels, a protein oxidation product, in KFs, which was consistent with De Felice et al.’s results. They also identified the reduced expression of Nrf2 in KFs. Similarly, Carney et al. [19] identified that ROS scavenging genes were downregulated at all time points during hypertrophic scar formation in a Duroc pig model, indicating that scar formation may be related to oxidative stress.

Adipose-derived mesenchymal stem cells (AD-MSCs) have shown prominence in the field of regenerative medicine because they are easier to obtain and have a wide variety of sources. AD-MSCs are currently considered potential therapeutic strategies for several diseases, particularly hypertrophic scars. An increasing number of studies have shown that AD-MSCs have a significant therapeutic effect on hypertrophic scars [20,21]; however, the specific mechanism is not clear. It has been confirmed that this kind of effect may be related to the TGF-β/Smad pathway, p38/MAPK pathway, etc. [22,23].

Notably, our study found that AD-MSCs could affect HSFs by promoting their apoptosis and inhibiting their proliferation, thus exerting their antifibrotic effects through in vivo and in vitro studies. In addition, we suggested that AD-MSCs caused apoptosis by downregulating the expression of Nrf2 in HSFs, leading to a reduced antioxidant enzyme expression and the accumulation of ROS.

## 2. Methods

### 2.1. Isolation and Culture of AD-MSCs

Human adipose tissue was obtained from patients who had undergone lipoplasty, and all enrolled patients signed the informed consent form to indicate their agreement and consent to the use of their adipose tissue in this study. The liposuction sites were bilateral thighs and buttocks. The adipose tissue was rinsed three times with phosphate-buffered saline (PBS, Solarbio, Beijing, China), and then 0.1% type I collagenase (Sigma–Aldrich, St. Louis, MO, USA) was used to digest the adipose tissue for 50 min. Then, the stromal vascular fraction was filtered through a 70 μm porous filter (Millipore, Hayward, CA, USA) after centrifugation at 2000 rpm for 10 min. After that, AD-MSCs were resuspended in Dulbecco’s modified Eagle medium: F-12 (DMEM/F-12, Gibco BRL, Waltham, MA, USA) containing 10% fetal bovine serum (FBS, Gibco BRL, NY, USA) and 1% penicillin–streptomycin (Solarbio, Beijing, China) at 37 °C in an incubator with 5% carbon dioxide (CO_2_). The medium was changed every two days. Cells in passage four were used in this experiment.

### 2.2. Flow Cytometry Used to Identify AD-MSCs

Flow cytometry was used to identify the phenotype of the cultured AD-MSCs. AD-MSCs were collected and washed three times in PBS and then were resuspended in PBS containing 1% bovine serum albumin (BSA, Invitrogen, Carlsbad, CA, USA). The cell suspension was incubated with fluorescein isothiocyanate (FITC)-conjugated antibodies against CD90 and CD34; phycoerythrin (PE)-conjugated antibodies against CD105, CD31, and CD45; and Brilliant Violet 421 (BV421)-conjugated antibodies against CD73 at 4 °C for 30 min in the dark. After washing twice, the cells were resuspended in 2% BSA and detected with a FACSCalibur instrument (BD Biosciences, San Jose, CA, USA). Data were analyzed using FlowJo software (Tree Star, Inc., Ashland, OR, USA).

### 2.3. Adipogenic and Osteogenic Differentiation

AD-MSCs were inoculated in six-well plates (Corning, Corning, NY, USA) at a cell density of 2 × 10^5^/well until the confluence of cells reached 80%. Adipogenic differentiation was proceeded using basic medium A containing 10% FBS, 1% penicillin–streptomycin, 1% glutamine, 0.2% insulin, 0.1% 3-isobutyl-1-methyl xanthine, 0.1% rosiglitazone, and 0.1% dexamethasone for 3 days and basic medium B containing 10% FBS, 1% penicillin–streptomycin, 1% glutamine, and 0.2% insulin for 1 day, and they alternated 4 times (Cyagen Biosciences, Inc., Suzhou, China, HUXMD-90031).

Osteogenic differentiation was proceeded using basic medium containing 10% FBS, 1% penicillin–streptomycin, 1% glutamine, 0.2% ascorbate, 1% β-glycerophosphate, and 0.01% dexamethasone for 3 weeks (Cyagen Biosciences, Inc., China, HUXMD-90021).

At the end of induction, 4% paraformaldehyde (Solarbio, Beijing, China) was used to immobilize the cells for 30 min, and Oil Red O and Alizarin Red S dye solutions were used to assess adipogenic and osteogenic differentiation according to the manufacturer’s instructions, respectively. The cells were observed under a microscope (Olympus, Tokyo, Japan) after staining.

### 2.4. Chondrogenic Differentiation

AD-MSCs were harvested and resuspended in a centrifuge tube at a cell density of 4 × 10^5^/tube. The medium contained 0.3% ascorbate, 0.01% dexamethasone, 1% insulin ferro-selenium transporter supplement, 0.1% sodium pyruvate, 0.1% proline, and 1% transforming growth factor-β3 (Cyagen Biosciences, Inc., China, HUXMD-90041)). The cells were cultured at 37 °C in 5% CO_2_ for 21 days.

After induction, 4% paraformaldehyde was used to immobilize the cartilage balls for 30 min at room temperature, and Alcian Blue staining was used to assess chondrogenic differentiation according to the manufacturer’s instructions. The sections were examined under the microscope.

### 2.5. HSF Isolation and Cultivation

Hypertrophic scar samples were obtained from patients who had undergone plastic surgery, and all enrolled patients signed the informed consent form to indicate their agreement and consent to the use of their hypertrophic scar tissue in this study. Finally, a total of 8 samples were collected, including five males and three females with an average age of 35.0 ± 11.7 years.

Dermal tissues were washed three times with PBS and then minced into pieces (~1 mm). Pieces were explanted to Dulbecco’s Modified Eagle Medium (DMEM, Gibco BRL, NY, USA) containing 10% FBS and 1% penicillin–streptomycin and were incubated at 37 °C in 5% CO_2_. The medium was changed every two days. After 7–10 days in the primary culture, the cells proliferated at the edge of the explanted tissues at which time we removed the explanted tissues. When the confluence of the cells reached 80%, the cells were passaged. Cells in passage four were used in this experiment.

### 2.6. Established Indirect Cocultivation System

HSFs were resuspended in DMEM/F-12 and inoculated into the lower chamber of a Transwell coculture plate. AD-MSCs were resuspended in DMEM/F-12 and inoculated into the upper chamber of the plate. Only the same amount of medium was added to the upper chamber in the control group. The number of inoculated AD-MSCs was adjusted to ensure that the final AD-MSC to HSF ratio was 0:1, 0.5:1, 1:1, and 2:1, respectively, with the 0:1 group being the control group.

A 6.5 mm Transwell with 0.4 µm sterile pore polycarbonate membrane insert (3413, Corning, USA) was used in Cell Counting Kit-8 (CCK-8) trial and immunofluorescence staining. A 24 mm Transwell with 0.4 µm sterile pore polycarbonate membrane insert (3450, Corning, USA) was used in the scratch assay and other experiments.

In the experiments using Transwell plates (3450, Corning, USA), the number of HSFs in the low chamber was 2 × 10^5^, and the number of AD-MSCs in the upper chamber was 10^5^, 2 × 10^5^, and 4 × 10^5^ for 0.5:1, 1:1, and 2:1 groups, respectively. As for the experiments using Transwell plates (3413, Corning, USA), the number of HSFs in the low chamber was 5 × 10^4^, and the number of AD-MSCs in the upper chamber was 2.5 × 10^4^, 5 × 10^4^, and 10^5^ for 0.5:1, 1:1, and 2:1 groups, respectively.

### 2.7. Cell Proliferation and Migration Assays

Cell proliferation was determined using Cell Counting Kit-8 (Beyotime Biotechnology, Shanghai, China). HSFs were collected and resuspended in DMEM/F-12 after culture with AD-MSCs for 24 h, 48 h, and 72 h, and the HSFs in each well were then divided equally into three portions and were inoculated in 96-well plates. Until the cells were attached to the dish entirely, 100 μL of DMEM/F-12 medium containing 10% CCK-8 solution was added to each well. The optical density at 450 nm was measured with a multiwell plate reader (Tecan, Morrisville, NC, USA).

The migration property was evaluated through scratch assay. HSFs were cultured in a six-well plate, and, when 70% confluence was reached, the cells were scratched with a pipet tip through the well bottom center. Additionally, AD-MSCs were added to the upper chamber of the plate as previously described. Images were taken using a microscope (Olympus, Tokyo, Japan) every 24 h. ImageJ software was used to measure the area and length of the scratches to calculate the average width of the scratches. The migration rate of the scratches was calculated as follows: migration rate (%) = *(W*_0_ − *W_t_)/W*_0_ *×* 100%, where *W*_0_ is the original width and *W_t_* is the remaining width at the measured time point.

### 2.8. Cell Cycle Assays

Cell cycle was determined through flow cytometry using a Cell Cycle Assay Kit (Beyotime Biotechnology, Shanghai, China) according to manufacturer’s instructions. Briefly, HSFs were harvested and fixed with 70% cold ethanol overnight at 4 °C. On second day, propidium iodide (PI) solution was added for DNA staining for 30 min at 37 °C, and the cells were detected with a FACSCalibur instrument (BD Biosciences, CA, USA). Data were analyzed using FlowJo software (Tree Star, Inc., Ashland, OR, USA).

### 2.9. Annexin V-Fluorescein Isothiocyanate (FITC) and Propidium Iodide (PI) Staining

Cell apoptosis was detected with an Annexin V-FITC PI Apoptosis Kit (BD Biosciences, CA, USA). After coculture for 24 h to 48 h, the HSFs were collected and resuspended in a flow tube with 1 × Annexin V Binding Buffer, and Annexin V-FITC and PI were added according to the instructions. After incubation for 15 min, the cells were detected with a FACSCalibur instrument (BD Biosciences, CA, USA). Data were analyzed using FlowJo software (Tree Star, Inc., Ashland, OR, USA).

### 2.10. Intracellular ROS Evaluation

Intracellular ROS were measured through flow cytometry using ROS Assay Kit (Beyotime Biotechnology, Beijing, China) with 2,7-dichlorofluorescein diacetate (DCFH-DA) as a fluorescent probe. After 48 h of coculture, HSFs were washed three times with PBS and were incubated with DCFH-DA for 30 min at 37 °C in the dark according to the instructions. The labelled cells were washed with PBS three times and evaluated immediately with a FACSCalibur instrument (BD Biosciences, CA, USA). Data were analyzed using FlowJo software (Tree Star, Inc., Ashland, OR, USA).

### 2.11. Total Superoxide Dismutase (T-SOD) Activity

A T-SOD Activity Assay Kit (Nanjing Jiancheng Bioengineering Institute, Nanjing, China) was used to detect the T-SOD activity of HSFs after 48 h of coculture based on the autoxidation of hydroxylamine. All procedures were carried out according to the instructions, and the developed color was measured at 550 nm using a multiwell plate reader (Tecan, USA).

### 2.12. Quantitative Real-Time Polymerase Chain Reaction (qRT–PCR)

Briefly, total RNA was extracted from HSFs after 48 h of coculture with AD-MSCs using TRIzol Reagent Kit (Invitrogen, Carlsbad, CA, USA). The concentration and purity of RNA was detected using a nanophotometer (Implen, Munich, Germany), and samples with A260/A280 value between 1.8 and 2.0 were considered to be of high purity. The RNA was reverse transcribed into complementary DNA using the Prime Script RT Reagent Kit (Takara, Tokyo, Japan). Quantitative PCR was performed using the CFX96^TM^ Real-Time System (Bio-Rad, Hercules, CA, USA) and SYBR Premix Ex Taq II (Takara, Beijing, China) in a 12 μL PCR solution. Primers were obtained from Takara Biotechnology. The primer pairs used for gene amplification were as follows. Nrf2: forward GTATGCAACAGGACATTGAGCAAG and reverse TGGAACCATGGTAGTCTCAACCAG. Keap1: forward CATCGGCATCGCCAACTTC and reverse ACCAGTTGGCAGTGGGACAG. NQO1: forward GGATTGGACCGAGCTGGAA and reverse GAAACACCCAGCCGTCAGCTA. GAPDH: forward GCACCGTCAAGGCTGAGAAC and reverse TGGTGAAGACGCCAGTGGA. The results were normalized against the mean Ct values of GAPDH using the ΔCt method as follows: ΔCt = Ct gene of interest—mean Ct (GAPDH). The fold increase was calculated as 2^−ΔΔCt^.

### 2.13. Western Blot

Radioimmunoprecipitation Assay Buffer (Beyotime Biotechnology, China) was used to lyse cells for 30 min on ice. The cells were then centrifuged at 12,000 × *g* at 4 °C to remove the cell debris. For tissue samples, after being minced into pieces, the tissue samples were immediately frozen in liquid nitrogen. Frozen tissues were collected in RIPA Assay Buffer with protease inhibitors (MedChemExpress, Monmouth Junction, NJ, USA) and phosphatase inhibitors (MedChemExpress, USA). Nuclear proteins were prepared with a Nuclear and Cytoplasmic Protein Extraction Kit (Beyotime Biotechnology, Shanghai, China). Samples (60 μg protein) were separated on 10% SDS–PAGE gels; transferred to a polyvinylidene fluoride membrane; blocked with 5% nonfat dried milk in TBST (10 mmol/L Tris, pH 7.5; 150 mmol/L NaCl, 0.05% Tween-20); and incubated with primary antibodies, including Nrf2 mouse monoclonal antibody (sc-365949, 1:2000, Santa Cruz Biotechnology, Paso Robles, CA, USA), HO-1 rabbit polyclonal antibody (10701-1-AP 1:1000, Proteintech, Wuhan, China), Bcl2 rabbit polyclonal antibody (12789-1-AP, 1:1000, Proteintech, Wuhan, China), BAX rabbit polyclonal antibody (50599-2-Ig, 1:1000, Proteintech, Wuhan, China), Caspase 3 rabbit polyclonal antibody (19677-1-AP, 1:1000, Proteintech, Wuhan, China), cleaved Caspase 3 rabbit monoclonal antibody (9664, 1:1000, Cell Signaling Technology, Danvers, MA, USA), and β-actin mouse monoclonal antibody (3700, 1:1000, Cell Signaling Technology, USA) at 4 °C overnight. After washing with TBST, the membranes were incubated with horseradish peroxidase-conjugated goat anti-rabbit or anti-mouse secondary antibodies (Epizyme Biotech, Shanghai, China). The immunoreactive bands were developed using an ECL Kit (Thermo Fisher Scientific, Waltham, MA, USA) and were detected with the Bio-Rad Molecular Imager Gel Doc ^TM^ XR+ (Bio-Rad, Hercules, CA, USA). Protein expression levels were quantified through densitometry analysis using ImageJ software (NIH, USA).

### 2.14. A Nude Mouse Model of In Vivo Transplantations of Hypertrophic Scars

In this study, we used twelve female nude mice (BALB/c-nu, 6–8 weeks old). Mice were obtained from SPF Biotechnology (Beijing, China) and maintained in the animal facility of the Fourth Medical Center of Chinese PLA General Hospital. Hypertrophic scar tissue sources are described above. All experimental procedures were performed following the regulations of the Institutional Animal Care and Use Committee.

Hypertrophic scar tissues were washed in PBS and divided into multiple small specimens (1.0 cm × 0.6 cm). After disinfection and anesthesia, four 1 cm incisions were made in the back skin of each mouse with scissors, and the scar tissues were implanted subcutaneously. The wound was sutured with 5–0 nylon thread and was left exposed. The transplanted scar tissues were stable after 4 weeks. Next, four hypertrophic scar tissue patches on the back of each mouse were treated four different ways. Blank group: no treatment. F12 group: 0.2 mL of DMEM/F-12. AD-MSC group: 0.2 mL of AD-MSCs (resuspended in DMEM/F-12; the total number of AD-MSCs was 2 × 10^6^). Triamcinolone acetonide group: 0.2 mL triamcinolone acetonide (triamcinolone suspended injection, 40 mg/mL per vial; Jida Chemical & Pharmaceutical Co., Kunming, China). For the AD-MSC group, cells were injected at four sites within the hypertrophic scar implant. The F12 group and the triamcinolone acetonide group underwent an equivalent procedure with DMEM. When the injection was successful, an elevation of the skin could be observed. Two weeks later, the injection was repeated. We collected tissues two weeks after each injection. After being weighed, the transplanted tissue collected was immediately stored in the −80 °C refrigerator for later use.

### 2.15. Immunofluorescence

For immunolabeling, HSFs after coculture or tissue samples of four groups were fixed in 4% paraformaldehyde for 15 min and were permeabilized in PBS with 0.1% Triton X-100 (HFH10, Invitrogen) for 10 min at room temperature. Nonspecific binding sites were blocked for 1 h with PBS containing 1% bovine serum albumin and 0.1% Tween 20. The fixed cells were incubated overnight at 4 °C with antibodies specific for Ki67 mouse monoclonal antibody (9449, 1:500, Cell Signaling Technology) and Nrf2 mouse monoclonal antibody (sc-365949, 1:100, Santa Cruz Biotechnology, CA, USA). Specific labelling was visualized using secondary antibodies conjugated with either Alexa 488 (ab150073, 1:200, Abcam) or Alexa 647 (ab150075, 1:200, Abcam). Nuclei were visualized through staining with DAPI (Thermo Fisher, Waltham, MA, USA). Images were acquired with a confocal microscope (SP8, Leica, Wetzlar, Germany).

### 2.16. Histological Analysis and TUNEL Assay

Tissue samples of all groups were excised, fixed in 4% paraformaldehyde, embedded in paraffin, sectioned at 5 μm thickness, mounted on slides, and stained with hematoxylin and eosin following the instructions. Masson’s staining was conducted using the ready-to-use kit (Trichrome Stain (Masson) Kit, HT15, Sigma–Aldrich). Briefly, the tissue was cut into 5 μm sections. Sections were immersed in Bouin’s solution (HT 10132, Sigma–Aldrich), stained in Weigert’s hematoxylin, incubated in phosphotungstic–phosphomolybdic acid, dyed with Aniline Blue, and fixed in 1% acetic acid. Then, the slides were rinsed in distilled water, dehydrated, observed, and photographed under a microscope (Olympus, Tokyo, Japan).

Apoptosis was analyzed on paraffinic hypertrophic scar tissue sections of the different groups with TUNEL Assay Kit (Millipore, USA). The slides were treated with 20 μg/mL of DNase-free proteinase K (AM2542, Invitrogen) at 37 °C for 30 min and were washed with PBS 3 times. Then, the slides were dyed using the TUNEL reaction solution prepared in a humid dark box for 1 h at 37 °C. After washing with PBS, the tissues were dyed using ProLong Diamond Antifade Mountant with DAPI (Thermo Fisher, Waltham, MA, USA). Images were acquired with the confocal microscope.

### 2.17. Statistical Analysis

The results are presented as average value ± standard deviation (SD). The data were analyzed using GraphPad Prism software 8.0 (GraphPad Software, Inc., San Diego, CA, USA). Student’s *t*-test was used for analysis between two groups. Differences with a *p*-value of <0.05 were considered statistically significant (* *p* < 0.05, ** *p* < 0.01, and *** *p* < 0.001).

## 3. Results

### 3.1. Characterization of AD-MSCs

**Figure 1:**
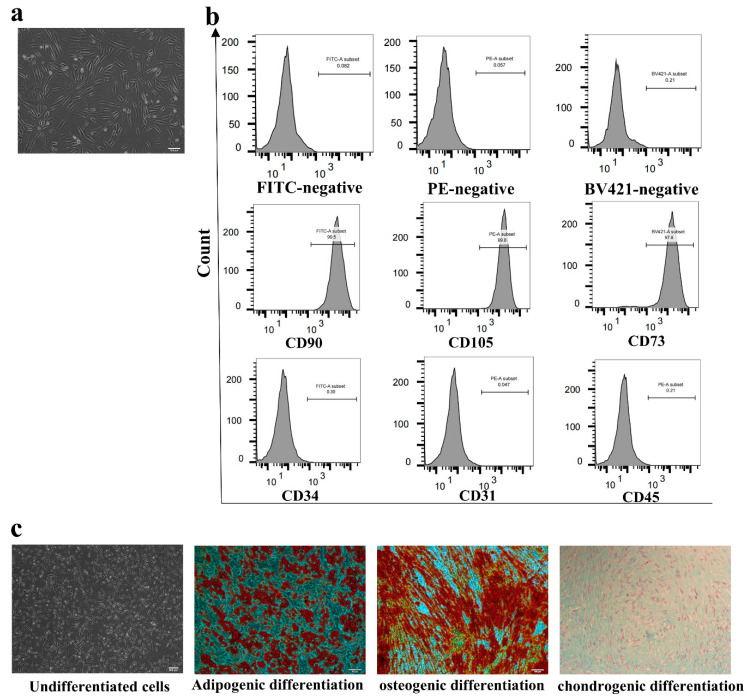
Characterization of human adipose-derived mesenchymal stem cells (AD-MSCs). (**a**) Photomicrograph of adherent AD-MSCs with spindle shapes on a cell culture dish. Scale bar = 100 μm. (**b**) Flow cytometric characterization of AD-MSCs. CD105, CD90, and CD73 were positive, while CD34, CD31, and CD45 were negatively expressed. (**c**) Multiple differentiation potential of AD-MSCs. The AD-MSCs were differentiated into matured adipocytes, osteocytes, and chondrocytes after incubation with adipogenic, osteogenic, or chondrogenic differentiation mediums at the times indicated, respectively. Adipocytes, osteocytes, and cartilage differentiated from AD-MSCs were determined through staining with Oil Red O, Alizarin Red and Alcian Blue, respectively. Scale bar = 200 μm.

The cultured primary and passaged AD-MSCs exhibited a spindle-shaped, fibroblast-like morphology as shown in Figure 1a. AD-MSCs were positive for mesenchymal stem cell markers CD105 (99.8%), CD90 (99.5%), and CD73 (97.8%) and negative for hematopoietic stem cell markers CD45 (0.21%), CD31 (0.04%), and CD34 (0.3%) as determined through flow cytometry (Figure 1b).

We examined the multipotential differentiation capacity of AD-MSCs using adipogenic, osteogenic, and chondrogenic assays. As was shown through Oil Red O staining, AD-MSCs developed an adipogenic phenotype after they were induced with the adipogenic medium for 21 days.

The Alizarin Red stain showed obvious orange calcium deposits and calcified nodules after it was induced with the osteogenic medium for 21 days. We also cultured AD-MSCs with the chondrogenic medium for 3 weeks and stained them with Alcian Blue, and the endo acid mucopolysaccharides were stained blue in the cartilage globules (Figure 1c). The results demonstrated that the isolated AD-MSCs showed typical AD-MSCs characteristics.

### 3.2. AD-MSCs Inhibited HSF Proliferation and Migration

**Figure 2:**
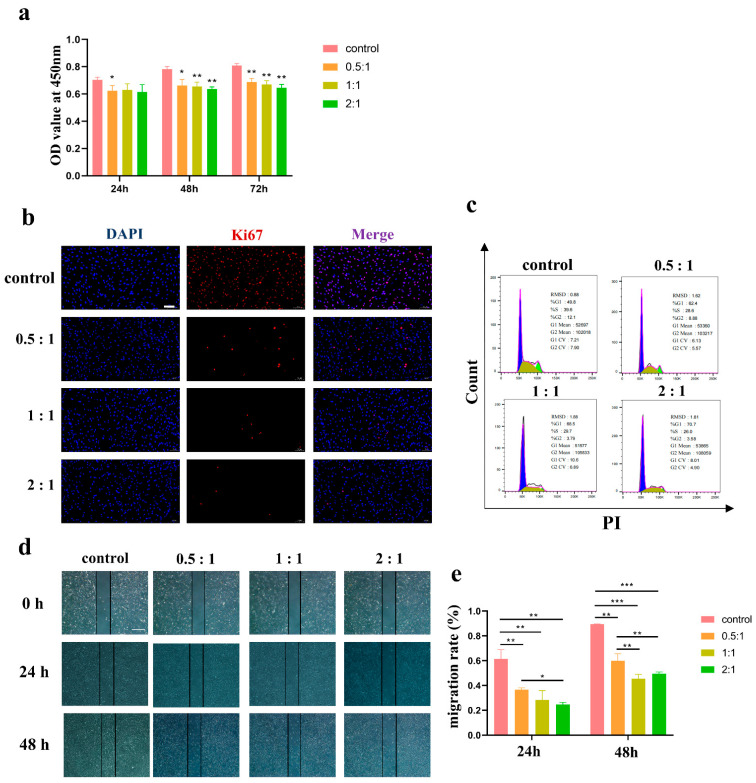
AD-MSCs inhibited proliferation and migration of HSFs. (**a**) Cell viability was detected with CCK-8 assay every 24 h after coculturing (n = 3). (**b**) Immunofluorescence staining used to determine the expression levels of Ki67 in HSFs of different groups, scale bar = 100 μm. (**c**) Flow cytometry was used to quantify cell cycle distribution in HSFs treated with AD-MSCs. (**d**,**e**) Representative images and the migration rate of the cell scratch assay treated with different concentrations of AD-MSCs, scale bar = 200 μm (n = 3). * *p* < 0.05, ** *p* < 0.01, and *** *p* < 0.001 vs. control.

In cases where we ensured that each group was initially inoculated with an equal number of cells, the CCK-8 assay reflected the proliferation of cells. The results showed that the number of HSFs in the control group were sharply increased when compared with the cocultured groups, indicating that significant vitality inhibition in HSFs was induced by AD-MSCs (Figure 2a). We further studied the expression of proliferation-related proteins in the control group and cocultured groups after they were cultured for 48 h using immunofluorescent staining. As shown in Figure 2b, the expression levels of the proliferation-related protein Ki67 in the HSFs of the coculture groups were significantly lower than those in the control group. Cell cycle experiments demonstrated consistent results, with the majority of the cells in the coculture group stalled in the G1 phase (Figure 2c). These results demonstrated that AD-MSCs inhibited the proliferation of HSFs.

To examine whether AD-MSCs affect the migration capacity of HSFs, we scratched the confluent cells to create a linear wound, and the cells were then cocultured for 48 h. The wound margin was marked on the picture, and the wound healing rate was quantified every 24 h after scratching. The results showed that a lower cell migration was observed in the presence of AD-MSCs as compared to that observed in the control group. At 24 h, the migration rate was 61.48% ± 7.42% in the control group, 36.61% ± 1.46% in the 0.5:1 group, and 28.39% ± 7.44% in the 1:1 group, whereas, in the 2:1 group, it was 24.72% ± 1.68%. Similarly, at 48 h, the migration rate was 89.34% ± 0.23% in the control group, 59.98% ± 5.58% in the 0.5:1 group, and 45.47% ± 3.49% in the 1:1 group, whereas in the 2:1 group, it was 49.45% ± 1.41%. At both time points, the coculture groups were statistically different from the control group (Figure 2d,e). The scratch assay demonstrated that AD-MSCs could significantly inhibit the rate of HSF wound closure compared with a normal medium in vitro.

### 3.3. AD-MSCs Promoted HSF Apoptosis

**Figure 3:**
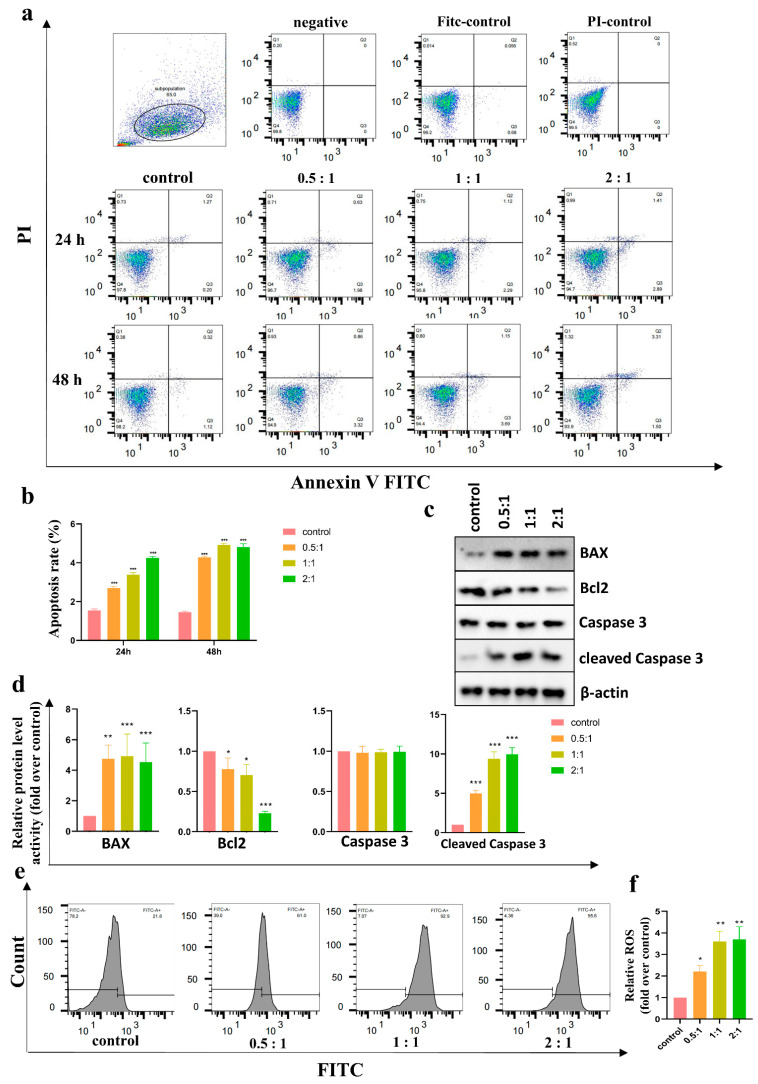
AD-MSCs induced apoptosis of HSFs. (**a**) Examination of apoptotic cells in HSFs after coculturing. The apoptosis of cells was assessed by FACS after 24 h and 48 h. (**b**) Quantitative analysis of the percentage of apoptotic cells as shown in a (n = 3). (**c**,**d**) The expression analysis of Bcl-2, Bax, Caspase 3, and cleaved Caspase 3 in HSFs of different groups after 48 h with immunoblotting assay (n = 3). (**e**,**f**) FACS detected the level of intracellular ROS of four groups after coculturing for 48 h. Quantitative analysis was shown in f (n = 3). * *p* < 0.05, ** *p* < 0.01, and *** *p* < 0.001 vs. control.

To investigate the biological effects of AD-MSCs on the apoptosis of HSF cells, we cocultured AD-MSCs with HSFs at different ratios (0:1, 0.5:1, 1:1, and 2:1) for 48 h. The 0:1 group was used as the control group. Flow cytometry analysis was performed every 24 h after culturing. As shown in Figure 3a,b, the apoptotic rate of HSFs cocultured with AD-MSCs were significantly increased in comparison to that of the control group. Moreover, the apoptosis rate of HSFs increased as the incubation time increased or the number of AD-MSCs increased. However, there was no statistical difference between the 1:1 and 2:1 groups at 48 h.

Additionally, the associated apoptotic proteins Bcl-2, Bax, Caspase 3, and cleaved Caspase 3 in HSFs were detected through Western blot. When compared with the control group, BAX and cleaved Caspase 3 levels were increased, and Bcl-2 levels were decreased in the cocultured groups (Figure 3c,d). (The full blots are shown in Supplementary Figure S3.)

### 3.4. AD-MSCs Increased the Accumulation of the ROS of HSFs

Intracellular ROS accumulation is a marker of oxidative stress which can lead to apoptosis. Flow cytometry analysis was performed to detect intracellular ROS. Results were expressed as a multiple of the control group. The relative accumulation of ROS in HSFs after the groups were cocultured with AD-MSCs for 48 h was increased compared with that in the control group (0.5:1: (2.21 ± 0.27), 1:1: (3.59 ± 0.48), 2:1: (3.70 ± 0.58), fold over control). Among the three cocultured groups, the ROS content in the 0.5:1 group was lower than that in the other two groups, and there was no statistical significance between the 1:1 group and the 2:1 group (Figure 3e,f).

### 3.5. Proliferation, Migration, and Apoptosis of HSFs after ROS Scavenging

To clarify whether the effect of AD-MSCs on HSFs was ROS-dependent, we added tempol (Sigma–Aldrich, St. Louis, MO, USA), an ROS scavenger, and repeated the proliferation, migration, and apoptosis assays.

**Figure 4:**
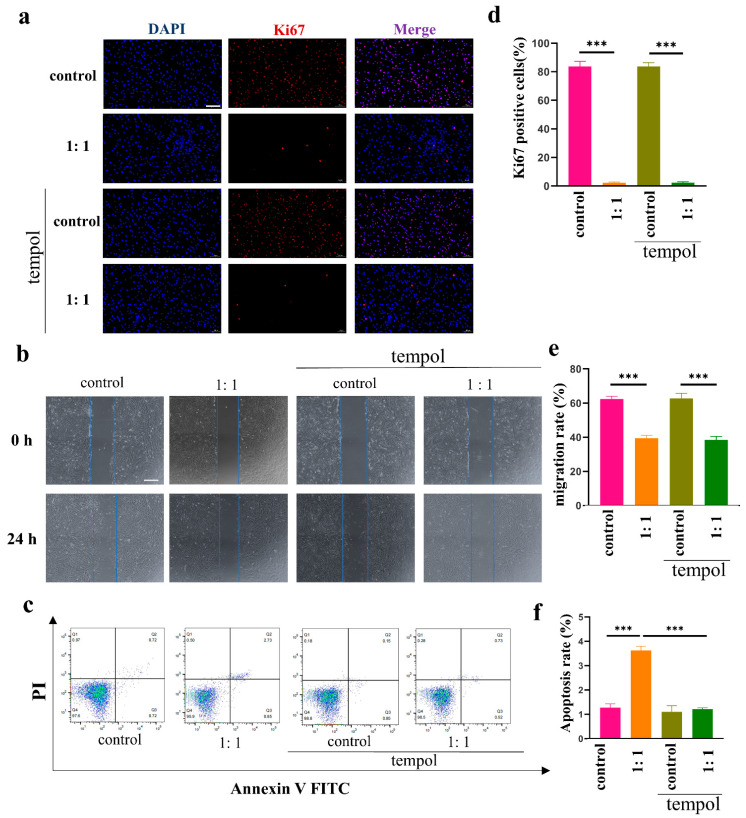
Proliferation, migration, and apoptosis of HSFs after ROS scavenging. (**a**) Immunofluorescence staining used to determine the expression levels of Ki67 in HSFs after coculturing with or without tempol, scale bar = 100 μm. (**b**) Representative images of the cell scratch assay of HSFs after coculturing treated with or without tempol, scale bar = 200 μm. (**c**) Examination of apoptotic cells in HSFs after coculturing with or without tempol. The apoptosis of cells was assessed through FACS after 24 h. (**d**) Quantitative analysis of the percentage of Ki67 positive cells as shown in a (n = 3). (**e**). Quantitative analysis of the migration rate in b (n = 3). (**f**) Quantitative analysis of the percentage of apoptotic cells as shown in c (n = 3).*** *p* < 0.001 vs. control.

It can be clearly seen from Figure 4a that the expression of Ki67 was significantly lower in the 1:1 group compared to the control group, which has been mentioned above. The expression of Ki67 was not significantly altered after the addition of tempol to the scavenged ROS. The results of the scratch assay and the proliferation assay were consistent, indicating that tempol did not change the effect of AD-MSCs on HSFs in terms of proliferation and migration (Figure 4b). However, the rate of apoptosis in HSFs was significantly lower in the coculture group after the scavenging of ROS, indicating that apoptosis was corrected (Figure 4c).

### 3.6. AD-MSCs Inhibited the Expression of Nrf2 and the Antioxidant Enzymes in HSFs

**Figure 5:**
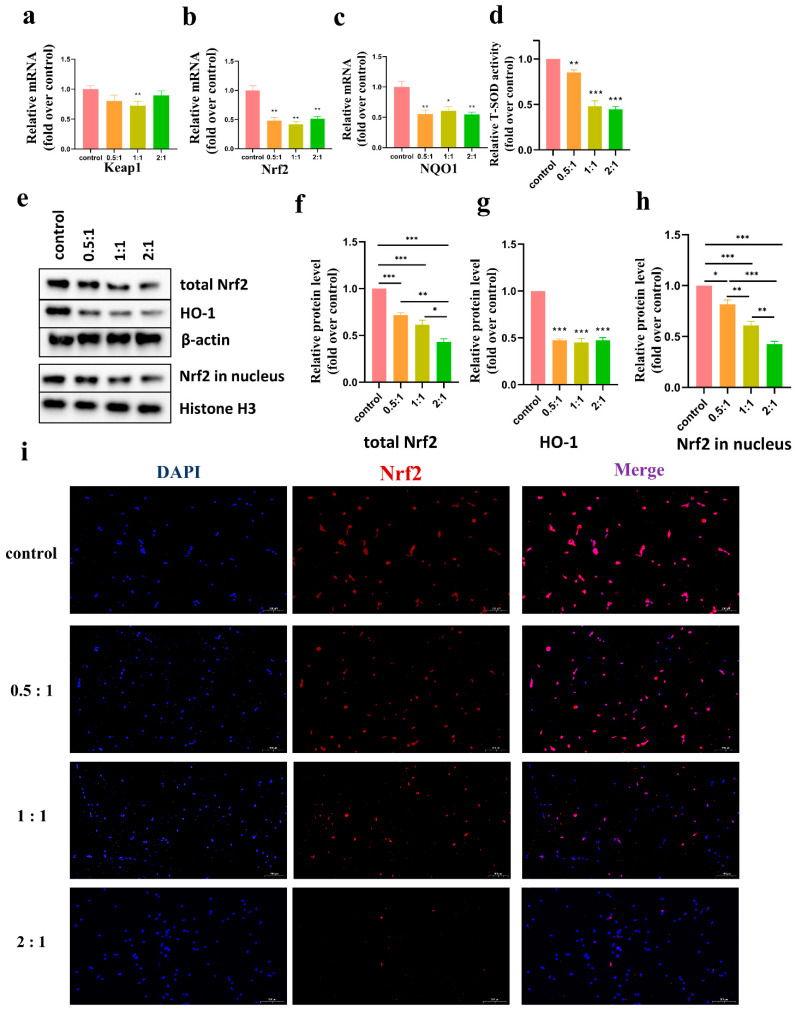
AD-MSCs inhibited the expression of Nrf2 and antioxidant enzymes. (**a**–**c**) The mRNA expression of KEAP1, Nrf2, and NQO1 in HSFs assessed after 48 h of coculturing was determined through RT-PCR. The graph represents the expression of Keap1, Nrf2, and NQO1 relative to that of GAPDH (n = 3). (**d**) T-SOD activity of HSFs was assessed after 48 h of coculturing, and the developed color was measured at 550 nm (n = 4). (**e**–**h**) Western blot assay for total Nrf2, HO-1, and Nrf2 in the nucleus after 48 h in HSFs of different groups (n = 3). (**i**) Immunofluorescence staining used to determine the expression levels of Nrf2 in HSFs of different groups after 48 h of coculturing, scale bar = 200 μm. * *p* < 0.05, ** *p* < 0.01, and *** *p* < 0.001 vs. control.

KEAP1/Nrf2 has been proven to regulate the gene expression of many antioxidant enzymes. As shown in Figure 5a–c, KEAP1, Nrf2, and NQO1 mRNA expression in HSFs after the groups were cocultured for 48 h was evaluated. The results demonstrated no significant difference in KEAP1 mRNA expression compared to the control group. In contrast, compared with the control group, Nrf2 and NQO1 mRNA expression were significantly decreased. As expected, Western blot analysis showed that the levels of total Nrf2, HO-1, and Nrf2 in the nucleus were decreased in HSFs cocultured with AD-MSCs compared to the control group (Figure 5e–h) (The entire blots are shown in supplementary Figure S3). Correspondingly, the results of immunofluorescence staining showed a significant decrease in Nrf2-positive cells in the coculture groups (Figure 5i).

SOD is capable of scavenging intracellular oxygen free radicals via the dismutation of superoxide radicals. We detected T-SOD activity in HSFs after the groups were cocultured with AD-MSCs for 48 h using a T-SOD Activity Assay Kit. The developed color was measured at 550 nm using an enzyme immunoassay analyzer, and the results were expressed as a multiple of the control group as shown in Figure 4d. After the groups were cocultured with AD-MSCs, their T-SOD activity was significantly lower than that in the control group, and there was no significant difference between the 1:1 group and 2:1 group (0.5:1: (0.85 ± 0.30), 1:1: (0.48 ± 0.05), 2:1: (0.45 ± 0.03), fold over control).

### 3.7. AD-MSCs Reduced the Weight of the Graft Scar and Rearranged the Collagen Fibers

**Figure 6:**
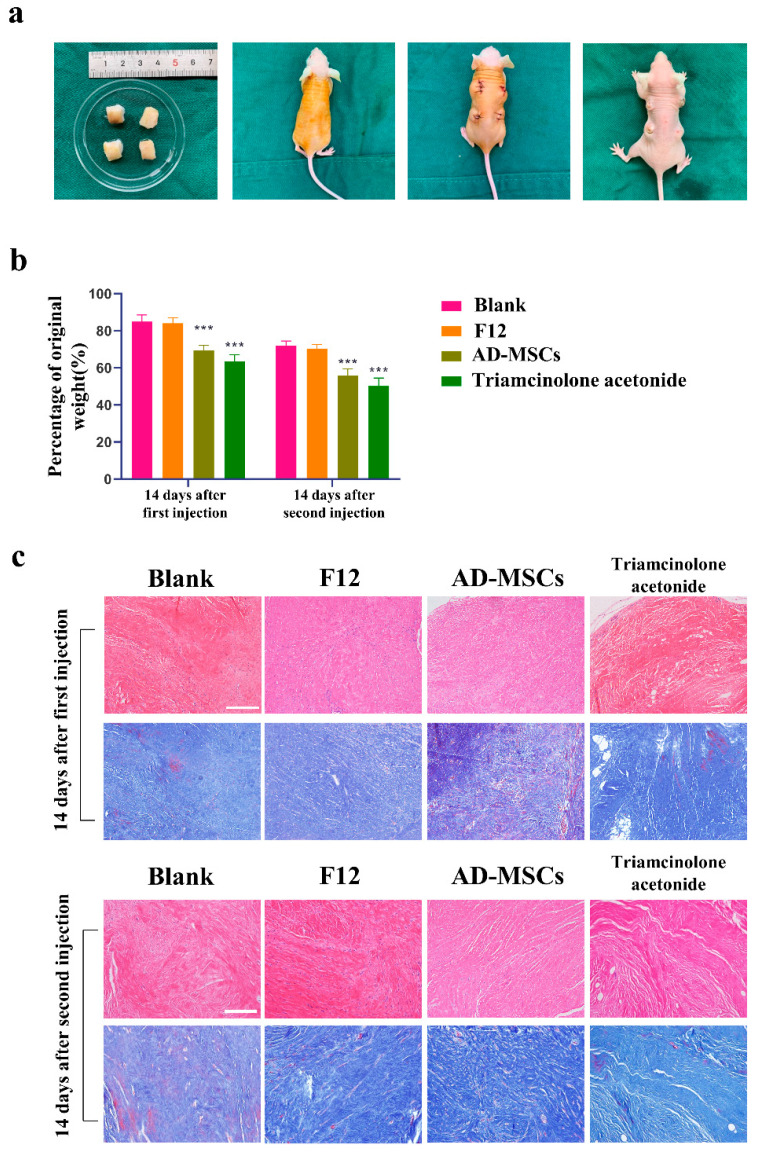
AD-MSCs reduced the weight of hypertrophic scar tissue transplanted in nude mice and rearranged collagen fibers. (**a**) The picture represents the process of animal model building. (**b**) Representative images show the histopathological changes and collagen deposition determined by hematoxylin and eosin and Masson’s trichrome, scale bar = 100 μm, respectively. (**c**) The bar chart shows the weight after treatment as a percentage of the original weight (n = 6). *** *p* < 0.001 vs. blank group.

To further verify the therapeutic effect of AD-MSCs on hypertrophic scars, we established a model of hypertrophic scar transplantation into nude mice (Figure 6a). Two weeks after treatment, we observed a significant reduction in the weight of the transplanted scar tissue in the AD-MSCs and triamcinolone acetonide groups compared to that in the blank group (Figure 6b). Additionally, hematoxylin and eosin staining and Masson’s staining showed thicker collagen fibers that exhibited disordered alignment in the multipolar, swirled, and nodular structures in the blank and F12 groups. The collagen fibers in the AD-MSC group were orderly arranged with a regular direction, and gaps between the collagen fibers were observed. Furthermore, the collagen fibers were loosely arranged in the triamcinolone acetonide group, and the gaps between the collagen fibers were more pronounced (Figure 6c).

### 3.8. AD-MSCs Promoted Apoptosis and Inhibited the Expression of Nrf2 In Vivo

**Figure 7:**
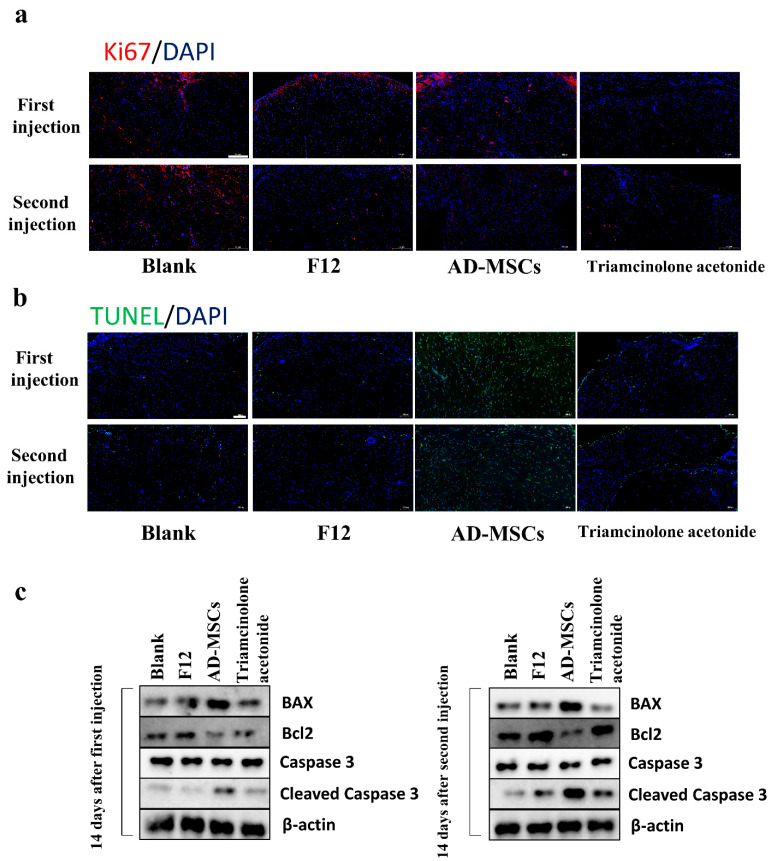
AD-MSCs induced apoptosis of hypertrophic scar tissue transplanted in nude mice. (**a**) Proliferation of cells was determined with immunofluorescence using antibodies against Ki67 in sections from transplanted tissues after the first and second injection of blank, F12, AD-MSCs, and triamcinolone acetonide groups. Scale bar = 200 μm. (**b**) Estimated apoptosis in sections from transplanted tissues after the first injection and second injection of blank, F12, AD-MSCs, and triamcinolone acetonide groups using the TUNEL assay. Scale bar = 100 μm. (**c**) Western blot analysis of Bcl-2, Bax, Caspase 3, and cleaved Caspase 3 protein levels in transplanted tissues after the first and second injection of blank, F12, AD-MSCs, and triamcinolone acetonide groups.

First, no significant Ki67-positive cells were observed in any of the four groups (Figure 7a). TUNEL staining showed that there were more apoptotic cells in the AD-MSC group compared to the other three groups (Figure 7b). Additionally, the associated apoptotic proteins, Bcl-2, Bax, Caspase 3, and cleaved Caspase 3, were detected in a Western blot. The BAX and cleaved Caspase 3 levels were increased, and the Bcl-2 levels were decreased in the AD-MSC group (Figure 7c) (The entire blots are shown in supplementary Figure S3).

**Figure 8:**
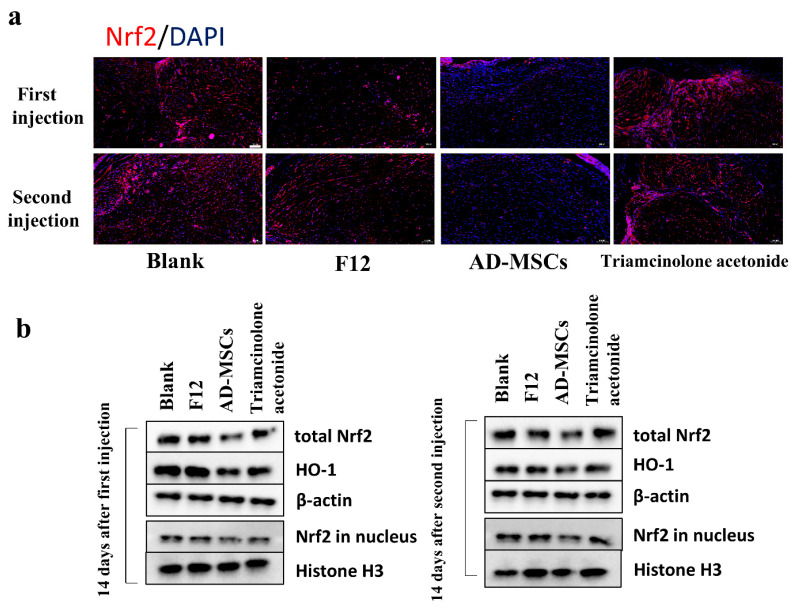
AD-MSCs inhibited the expression of Nrf2 and antioxidant enzymes in hypertrophic scar tissue transplanted in nude mice. (**a**) Levels of Nrf2 expression were determined through immunofluorescence in sections from transplanted tissues after the first and second injection of blank, F12, AD-MSCs, and triamcinolone acetonide groups. Scale bar = 100 μm. (**b**) Western blot assay for expression of total Nrf2, HO-1, and Nrf2 in nucleus in hypertrophic scar tissue transplanted in nude mice of different groups.

We also detected the expression of Nrf2 and HO-1 in the transplanted hypertrophic scar tissues after treatment to identify changes in the Nrf2 and antioxidant enzymes in vivo. As expected, the changes in these substances were consistent with those seen in vitro. As shown in Figure 8a,b, the expression of Nrf2 was lower in the tissues after AD-MSC injection than in the other three groups, and the expression of HO-1 also changed accordingly.

## 4. Discussion

The incidence of hypertrophic scars is particularly high, especially after burns and trauma. For patients, scar contracture, deformity, dysfunction, and accompanying itching are the biggest obstacles that hinder their return to society and their buildup of confidence in life. Although researchers have explored the pathogenesis of hypertrophic scars for decades, it remains unclear. It has been confirmed that many factors in the process of wound healing may be involved, such as the PI3K/AKT signaling pathway and the TGF-β/Smad signaling pathway, which regulates the process of fibrosis [4,24].

Oxidative stress may affect the process of fibrosis. Lee et al. [18] identified a reduced expression of Nrf2 and a higher level of oxidative stress in KFs, and they also demonstrated that Nrf2 could regulate Bcl2 expression. Studies have shown that ROS, such as hydrogen peroxide, can activate the TGF-β/Smad pathway [25], which may be one of the pathogeneses of hypertrophic scars. In addition, previous researchers have examined the expression of 25 antioxidant enzymes during the process of hypertrophic scar formation in pigs, including catalase and glutathione s-transferase, and found that from the time of wound healing to the formation of the hypertrophic scars, the expression levels of the 25 enzymes were all lower than those of the control group [19].

There are many treatments for hypertrophic scars, including surgical resection, lasers, and cryotherapy, but all of these treatments have side effects that need to be solved [26]. In recent years, with the deepening of the research on mesenchymal stem cells, some researchers have applied AD-MSCs to treat hypertrophic scars and have found that AD-MSCs can suppress hypertrophic scar fibrosis [23,27]. The mechanism may be that AD-MSCs regulate the p38/MAPK signaling pathway. Nevertheless, the concrete mechanism still needs to be explored.

We successfully extracted and cultured human AD-MSCs. According to previous studies [28], AD-MSCs should express several mesenchymal markers. After identification, it was found that the markers of mesenchymal stem cells, such as CD105, CD90, and CD73, were highly expressed, while other cell markers, such as CD45, CD31, and CD34, were negative. In addition, AD-MSCs also had adipogenic, osteogenic, and chondrogenic differentiation abilities, which was consistent with previous studies.

HSFs were extracted from hypertrophic scar tissues. According to the published literature, although HSFs have the same spindle-shaped morphology as normal fibroblasts, their gene expression and biological behavior are different; they produce more ECM and proliferate faster [29]. To identify whether the fibroblasts we extracted were HSFs, we collected normal skin tissue and extracted its fibroblasts. Then the expression of collagen I and fibronectin were detected through immunofluorescence. As shown in supplementary Figure S1, the expression of collagen I and fibronectin was higher than that in normal fibroblasts, which was consistent with the literature [30].

The indirect coculture of two types of cells using a Transwell is a method accepted by researchers. Transwell plates with 0.4 µm pore polycarbonate membranes were used in our study in which cells could not pass through the micropores. We assessed the cell viability, surface markers, and differentiation potential of AD-MSCs after coculturing for 48 h. As shown in supplementary Figure S2, no significant changes were seen in the cell viability or surface markers of AD-MSCs after indirect coculture. In addition to this, after the induction culture, AD-MSCs could still differentiate into bone, adipose, and cartilage.

In our study, after the groups were cocultured at different concentrations, AD-MSCs significantly inhibited the proliferation and migration of HSFs as shown in Figure 2. Similarly, Wang et al. [31] evaluated the effect of AD-MSCs on the proliferation and migration capacity of KFs and found that the proliferation and migration of KFs was inhibited. They supposed that the effect of AD-MSCs on the proliferation and migration of KFs might be related to the PI3K/AKT and MAPK signaling pathway.

Previous studies have shown that AD-MSCs induce apoptosis and inhibit proliferation in KFs through paracrine effects [32]. Our team’s previous studies came to the same conclusion [33]. We wondered whether AD-MSCs induce apoptosis in HSFs, so we detected apoptosis in HSFs after coculturing with AD-MSCs using an Annexin V-FITC PI Apoptosis Kit. The results showed that AD-MSCs promoted HSF apoptosis, and the apoptosis rate of HSFs increased as the incubation time increased or the number of AD-MSCs increased. Moreover, the reduced expression of Bcl2 and the elevated expression of BAX and cleaved Caspase 3 also demonstrated the apoptosis of HSFs after coculture.

It is well known that an increased intracellular accumulation of ROS can lead to apoptosis [34]. Flow cytometry was used to detect the intracellular ROS content in the HSFs after coculture. Unsurprisingly, the ROS content in the coculture groups was significantly higher than that in the control group. To figure out whether the elevated intracellular ROS were related to the culture medium, we assayed the level of hydrogen peroxide in the medium of both the upper and lower chamber of the Transwell coculture plate and found that no culture medium contained hydrogen peroxide (< 1 μM). Therefore, we speculated that the accumulation of intracellular ROS might be caused by a decrease in antioxidant enzymes.

To figure out whether the effect of AD-MSCs on HSFs is ROS-dependent, we repeated the proliferation, migration, and apoptosis assays by adding tempol and a stable, effective ROS scavenger. It was clear that tempol corrected the apoptosis-promoting effect of AD-MSCs on HSFs, indicating that the effect was ROS-dependent. In contrast, tempol did not affect the inhibitory effect of AD-MSCs on the proliferation and migration of HSFs, indicating that these effects were not ROS-dependent. To the best of our knowledge, AD-MSCs can inhibit the PI3K/AKT and MAPK signaling pathways in keloid-derived fibroblasts, which are closely related to cell proliferation and migration [31]. The PI3K/AKT and MAPK signaling pathways are not ROS-dependent, and we conjectured that this was the underlying reason why AD-MSCs inhibited the proliferation and migration of HSFs.

We detected the levels of various antioxidant enzymes using different methods, including T-SOD, HO-1, and NQO1 and found that the level of these enzymes decreased as shown in Figure 5. The expression of antioxidant enzymes in vivo was mainly regulated by the KEAP1/Nrf2 signaling pathway. Nrf2 bound to KEAP1 in the cytoplasm under normal conditions. During intracellular ROS accumulation, Nrf2 dissociated from KEAP1 and was transferred into the nucleus to regulate the expression of antioxidant enzymes. As shown in Figure 5, after coculture, KEAP1 mRNA expression in HSFs was not significantly altered compared to that of the control group. This suggested that AD-MSCs may not affect the expression of KEAP1. However, our results showed a significant reduction in Nrf2 expression, both in the total Nrf2 content and the content in the nucleus. We speculated that the content reduction in the nucleus might have resulted from the reduction in the total Nrf2 content. However, it was not clear why the level of Nrf2 decreased. We understood that the PI3K/Akt signaling pathway is an important activator of Nrf2 [35,36]. Wang et al. [31] found that AD-MSCs could reduce the phosphorylated levels of Akt in KFs. Therefore, we assumed that the decrease in Nrf2 may have been related to the downregulation of Akt. It requires further research to verify the relationship.

To further verify the therapeutic effect of AD-MSCs on hypertrophic scars, we established a hypertrophic scar transplantation model based on the absence of thymic immunity in nude mice. The nude mouse is homozygous null for the Foxn1 gene, resulting in a lack of thymic epithelium and, as a consequence, a lack of T cells [37,38,39]. We believed that this animal model could better represent human hypertrophic scar characteristics compared to the rabbit ear scar model. We examined cell proliferation through immunofluorescence staining and found almost no proliferating cells. This may be because the cells in the transplanted scar tissue did not proliferate. We also observed a reduction in the weight of the transplanted tissue. In addition to this, we found that AD-MSCs could rearrange collagen fibers. Consistent with the results of the in vitro experiments, AD-MSCs resulted in increased apoptosis and the significantly reduced expression of Nrf2 and antioxidant enzymes in hypertrophic scar tissues.

Interestingly, in wound healing studies, researchers have shown that AD-MSCs promote the proliferation; migration; and, what’s more, inhibited apoptosis of fibroblasts derived from normal skin tissue [40,41]. Wound healing is a complex process that involves a variety of cells and cytokines. He et al. [42] found that AD-MSCs activate the Wnt/catenin signaling pathway in HaCaT cells and dermal fibroblasts, thereby promoting wound healing. The same conclusion was reached in a study by Ma et al. [43]. Zhang et al. [44] found that AD-MSCs can promote dermal fibroblast proliferation and migration and optimize collagen deposition via the PI3K/Akt signaling pathway to accelerate wound healing further. Researchers have also shown that AD-MSCs can promote wound angiogenesis to promote wound healing [45]. These studies suggest that AD-MSCs play an essential role in promoting fibrosis. However, our study and other studies of hypertrophic scars and keloids confirmed the antifibrotic effect of AD-MSCs [31,33]. We supposed that the reasons for this phenomenon might be attributed, to some extent, to the distinct expression of proliferation-, migration-, and apoptosis-related genes in the different organizational sources of fibroblasts. Abnormal gene expression levels may have different responses to AD-MSCs, leading to changes in the expression of other signaling pathways and ultimately affecting biological activity. In addition, the medium and additives required for a cell culture, the concentration of the cells cocultured, and the source of the specimens may also affect the results.

In summary, we showed that AD-MSCs are effective in hypertrophic scar treatment due to the reduction of scar weight and the promotion of collagen fiber remodeling and rearrangement. AD-MSCs inhibited the proliferation and migration of HSFs and promoted apoptosis. This was because they inhibited Nrf2 expression, leading to a reduction in antioxidant enzymes and an accumulation of ROS.

## 5. Conclusions

Our experiments revealed the therapeutic effects of AD-MSCs on hypertrophic scars and found that AD-MSCs inhibit the biological activity of HSFs. More importantly, AD-MSCs could downregulate the expression of Nrf2 in HSFs, resulting in a reduction in the expression of antioxidant enzymes and an accumulation of intracellular ROS, eventually activating the apoptosis program. We suspected that the downregulation of Nrf2 played an essential role in mediating AD-MSC-induced HSF apoptosis and antiproliferation effects. This pathway may act as a critical contributor to the mechanism of the antifibrotic effect of AD-MSCs.

## Data Availability

Supporting data can be obtained from the corresponding author.

## Supplementary Materials

The following supporting information can be downloaded at: https://www.mdpi.com/article/10.3390/cells11244024/s1, Figure S1: Differences between normal skin fibroblasts and hypertrophic scar fibroblasts; Figure S2: Characterization of AD-MSCs after coculturing with HSFs; Figure S3: Full bolts of the cropped bands which were shown in the article.

## Abbreviations

AD-MSCs: adipose-derived mesenchymal stem cells. qRT–PCR: quantitative real-time polymerase chain reaction. HSFs: hypertrophic scar fibroblasts. ROS: reactive oxygen species. Nrf2: nuclear factor erythroid-2-related factor 2. HO-1: heme oxygenase 1. Bcl2: B-cell lymphoma 2. BAX: Bcl2-associated X. ECM: extracellular matrix. SOD: superoxide dismutase. NQO1:NAD(P)H: quinone oxidoreductase 1. KFs: keloid fibroblasts. PBS: phosphate-buffered saline. FBS: fetal bovine serum. FITC: fluorescein isothiocyanate. PI: Propidium Iodide.
