# Supplementary material for: Adipose-Derived Mesenchymal Stem Cells Alleviate Hypertrophic Scar by Inhibiting Bioactivity and Inducing Apoptosis in Hypertrophic Scar Fibroblasts
Source: Cells. 2022 Dec 12;11(24):4024. doi: 10.3390/cells11244024 (PMC9776926; doi:10.3390/cells11244024)
Supplement: Supplementary file 1 [file cells-11-04024-s001.zip › cells-2058614-supplementary.pdf]

Supplementary materials

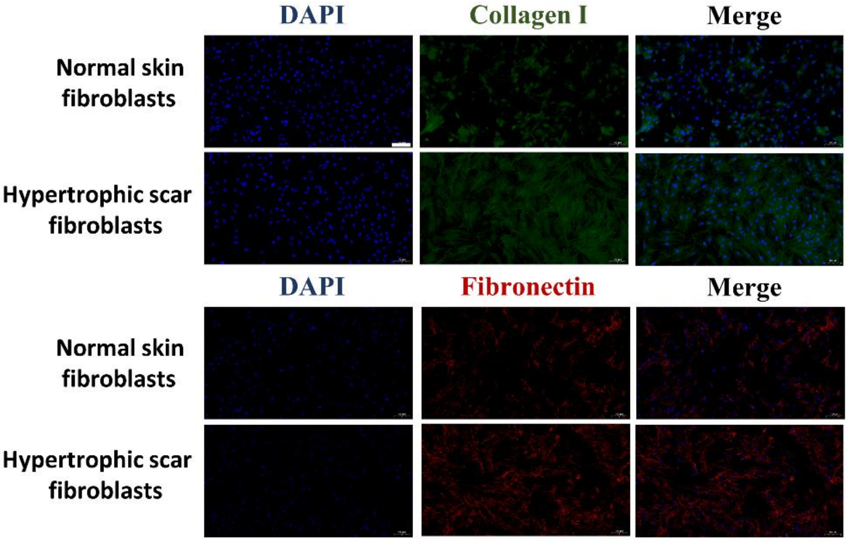

Figure S1. Differences between normal skin fibroblasts and hypertrophic scar fibroblasts. Cells was determined by immunofluorescence using antibodies against collagen I and fibronectin.

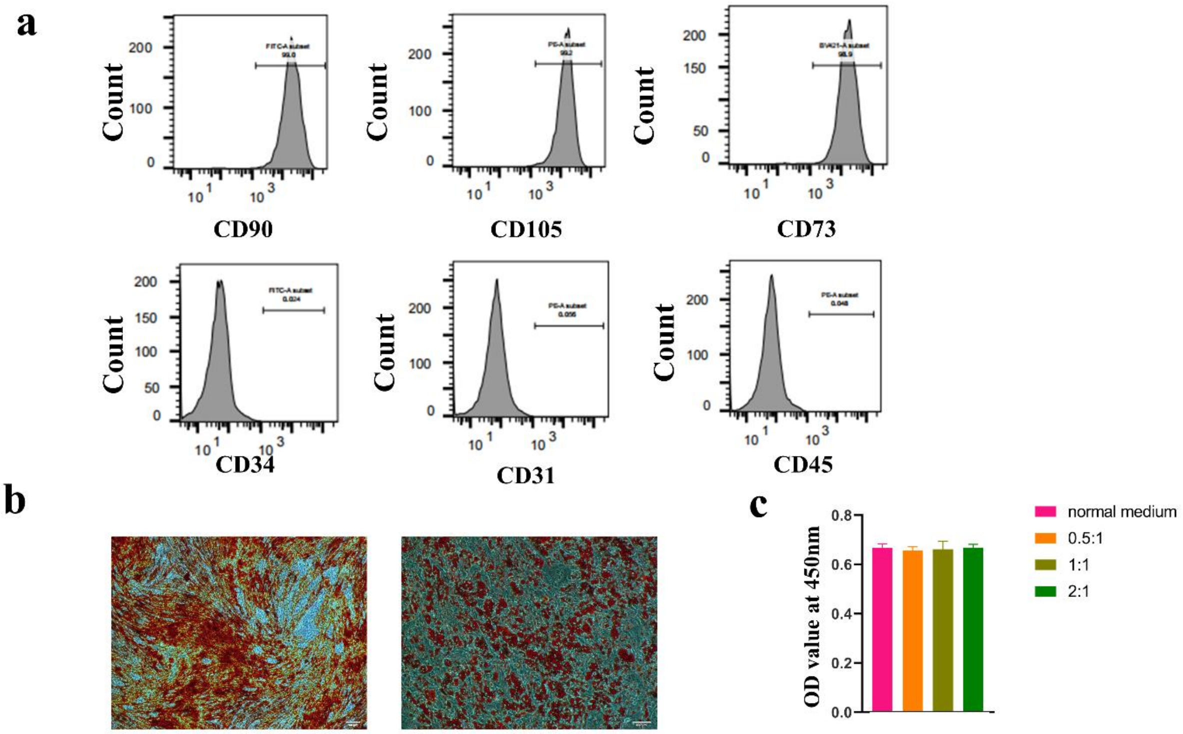

Figure S2. Characterization of AD-MSCs after coculturing with HSFs. a. Flow cytometric characterization of AD-MSCs. CD105, CD90 and CD73 were positive, while CD34, CD31 and CD45 were negatively expressed, which was not changed. b. The AD-MSCs could differentiate into matured adipocytes and osteocytes a after coculturing with HSFs. c. CCK-8 assay showed that, after coculturing, the proliferation of AD-MSCs had not changed.

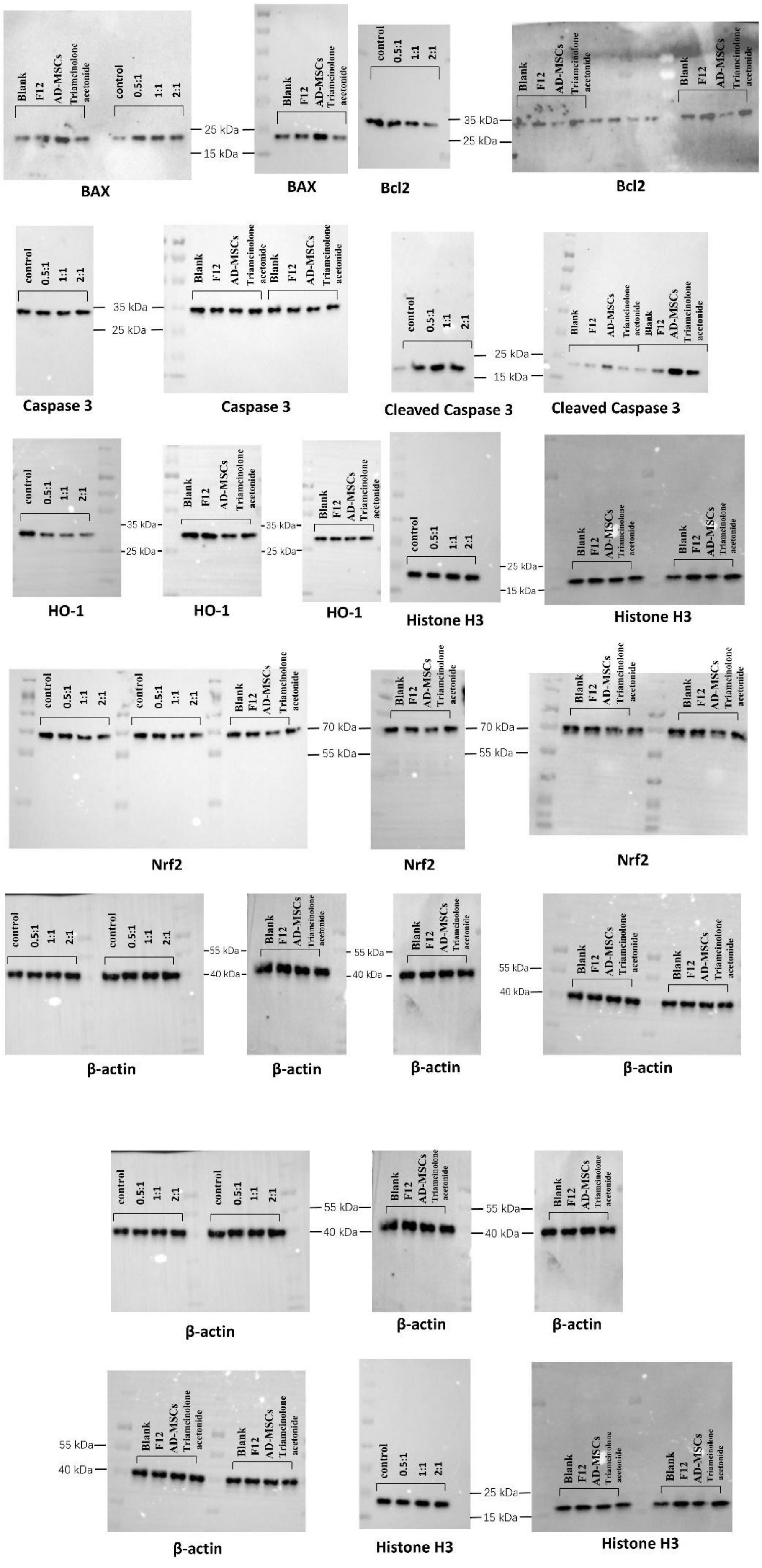
Blank
F12
AD-MSCs
Triamcinolone acetonide
control
0.5:1
1:1
2:1
25 kDa
15 kDa
35 kDa
70 kDa
55 kDa
40 kDa
BAX
BAX
Bcl2
Bcl2
Caspase 3
Caspase 3
Cleaved Caspase 3
Cleaved Caspase 3
HO-1
HO-1
HO-1
Histone H3
Histone H3
Nrf2
Nrf2
Nrf2
β-actin
β-actin
β-actin
β-actin
β-actin
β-actin
β-actin
β-actin
Histone H3
Histone H3

Figure S3. Full bolts of the cropped bands which were shown in the article.
